# Use of Point-of-Care Ultrasound by Non-Physicians to Assess Respiratory Distress in the Out-of-Hospital Environment: A Scoping Review

**DOI:** 10.1017/S1049023X22000711

**Published:** 2022-08

**Authors:** Jake K. Donovan, Samuel O. Burton, Samuel L. Jones, Benjamin N. Meadley

**Affiliations:** 1. Ambulance Victoria, Doncaster, Victoria, Australia; 2. Department of Paramedicine, Monash University, Frankston, Victoria, Australia

**Keywords:** interstitial syndrome, lung ultrasound, paramedic, respiratory failure, thoracic ultrasound

## Abstract

**Background:**

The use of ultrasound in the out-of-hospital environment is increasingly feasible. The potential uses for point-of-care ultrasound (POCUS) by paramedics are many, but have historically been limited to traumatic indications. This study utilized a scoping review methodology to map the evidence for the use of POCUS by paramedics to assess respiratory distress and to gain a broader understanding of the topic.

**Methods:**

Databases Ovid MEDLINE, EMBASE, CINAHL Plus, and PUBMED were searched from January 1, 1990 through April 14, 2021. Google Scholar was searched, and reference lists of relevant papers were examined to identify additional studies. Articles were included if they reported on out-of-hospital POCUS performed by non-physicians for non-traumatic respiratory distress.

**Results:**

A total of 591 unique articles were identified, of which seven articles met the inclusion criteria. The articles reported various different scan protocols and, with one exception, suffered from low enrolments and low participation. Most articles reported that non-physician-performed ultrasound was feasible. Articles reported moderate to high levels of agreement between paramedics and expert reviewers for scan interpretation in most studies.

**Conclusion:**

Paramedics and emergency medical technicians (EMTs) have demonstrated the feasibility of lung ultrasound in the out-of-hospital environment. Further research should investigate the utility of standardized education and scanning protocols in paramedic-performed lung ultrasound for the differentiation of respiratory distress and the implications for patient outcomes.

## Background

Out-of-hospital care is a rapidly evolving area of the health system in Australia.^1^ The provision of this care is primarily provided by paramedics and ambulance officers with significant variations in scope of practice and education.^2^ Respiratory distress is a frequent presentation in the out-of-hospital environment.^3,4^ These presentations are characterized by a high degree of diagnostic uncertainty as well as being potentially life-threatening.^3^ In the out-of-hospital setting, paramedics rely on clinical examination, history taking, and lung auscultation to differentiate causes of respiratory distress. The diagnostic accuracy of chest auscultation for the differentiation of respiratory complaints is low.^5^ Incorrect diagnosis and management of respiratory distress may result in a worse outcome for the patient.^6^

The first documented uses of ultrasound by physicians in emergency medicine date back to the 1990s.^7^ Paramedics have been utilizing point-of-care ultrasound (POCUS) in several jurisdictions for various indications for more than a decade.^8–10^ The most prevalent use of POCUS by paramedics is for the extended Focused Abdominal Scan in Trauma (eFAST).^8,9^ However, the benefits of POCUS for both assessment and procedural success extend to other clinical areas. For clinicians with even minimal experience in POCUS, lung ultrasound may be a quick and straightforward exam allowing for rapid and conclusive differentiation of respiratory complaints.

Signs on ultrasound images include lung sliding, which is the movement of the visceral and parietal pleura against each other and can be recognized by a hyperechoic shimmering line. A-lines are reverberation artefacts represented as echogenic horizontal lines and are indicative of normal lungs or a bronchospastic pathology. Additionally, B-lines are vertical lines originating from the pleura and are representative of fluid in the lungs.^4^ Finally, C-lines are characterized by a thickened irregular pleural line and are representative of pneumonia.

Lung ultrasound has been demonstrated to have a high sensitivity and specificity for differentiating respiratory pathologies and has been validated in the in-hospital setting.^11,12^ However, the available literature on out-of-hospital lung ultrasound for respiratory distress is mostly limited to physician-led systems.^13–15^ There have been a number of systematic reviews that focus on the in-hospital use of lung ultrasound,^12,16,17^ but also refer to or directly consider the out-of-hospital context. The advent of smaller and less costly ultrasound devices coupled with increased utilization of POCUS has the potential to increase the scope of indication for POCUS and improve the diagnostic accuracy of a range of clinical conditions in the out-of-hospital setting. There is little evidence regarding paramedic ability to utilize POCUS to acquire and interpret scans to differentiate causes of respiratory distress. In this study, a scoping review methodology was employed to map the evidence for the use of POCUS by paramedics to assess respiratory distress and to gain a broader understanding of the topic.

## Methods

This study aimed to map the literature relating to paramedic use of lung ultrasound for patients with respiratory distress for non-traumatic conditions. To compile the available evidence in the field and identify and analyze knowledge gaps, the methods described in the Joanna Briggs Institute (JBI; Adelaide, Australia) manual for evidence synthesis were utilized.^18^ The six-stage scoping review framework recommended by Levac, et al was also used.^19^

The research question for this review was: “Can paramedics acquire and interpret a lung ultrasound scan using a handheld ultrasound device to differentiate causes of respiratory distress in the out-of-hospital setting?” A search was conducted of two online databases relevant to the topic: Ovid MEDLINE (US National Library of Medicine, National Institutes of Health; Bethesda, Maryland USA) and EMBASE (Elsevier; Amsterdam, Netherlands). An analysis of the keywords and index terms of the retrieved papers was undertaken and then utilized in a second search of Ovid MEDLINE, EMBASE, CINAHL Plus (EBSCO Information Services; Ipswich, Massachusetts USA), and PUBMED (National Center for Biotechnology Information, National Institutes of Health; Bethesda, Maryland USA). The reference lists of the articles selected for full-text analysis were examined for further studies. The searches were limited to the dates January 1, 1990 - April 14, 2021. A further search was conducted using Google Scholar (Google Inc.; Mountain View, California USA) to identify any missed articles during the database search. The search strategy included Medical Subject Headings (MeSH terms) and keywords relevant to out-of-hospital care, paramedics, and lung ultrasound. Selected items were also incorporated from the paramedic literature search filter by Olaussen, et al.^20^ These terms were combined via Boolean operators during the database searches utilizing the Population/Concept/Context (PCC) format (Table 1).

**Table 1.** Summary of Population, Concept, and Context (PCC) Search Terms

PCC Element	Definition	Search Term
**Population**	Important characteristics of participants: ● Adults >18 years ● Respiratory distress	● Respiratory distress.mp ● Respiratory failure.mp ● Dyspnoea.mp ● Dyspnea.mp ● Interstitial syndrome.mp ● COPD.mp ● Chronic obstructive pulmonary disease.mp ● Pneumothorax.mp ● Pleural effusion.mp ● Consolidation.mp
**Concept**	Interventions/phenomena of interest/outcomes: ● Lung ultrasound ● Interpretation ● Image quality	● Lung Ultraso*.mp ● Thoracic ultraso*.mp ● Chest ultraso*.mp ● Sonography.mp ● Ultraso*.mp ● Diagnostic imaging.mp ● POCUS.mp ● Prehospital ultraso*.mp ● Portable ultraso*.mp ● Emergency ultraso*.mp
**Context**	Details about the setting: ● Out-of-hospital setting ● Paramedics	● Emergency medical technicians.sh ● Emergency services.sh ● Emergency medical services.sh ● Ambulances.sh ● Out-of-hospital.tw
		● Pre-hospital.tw ● Pre-hospital.tw ● Paramedic*.tw ● EMT.tw ● EMS.tw ● Air ambulance.tw ● Ambulance.tw ● First responder*.tw ● Emergency medical technicians.tw ● HEMS.tw ● Field triage.tw

**Figure 1.**
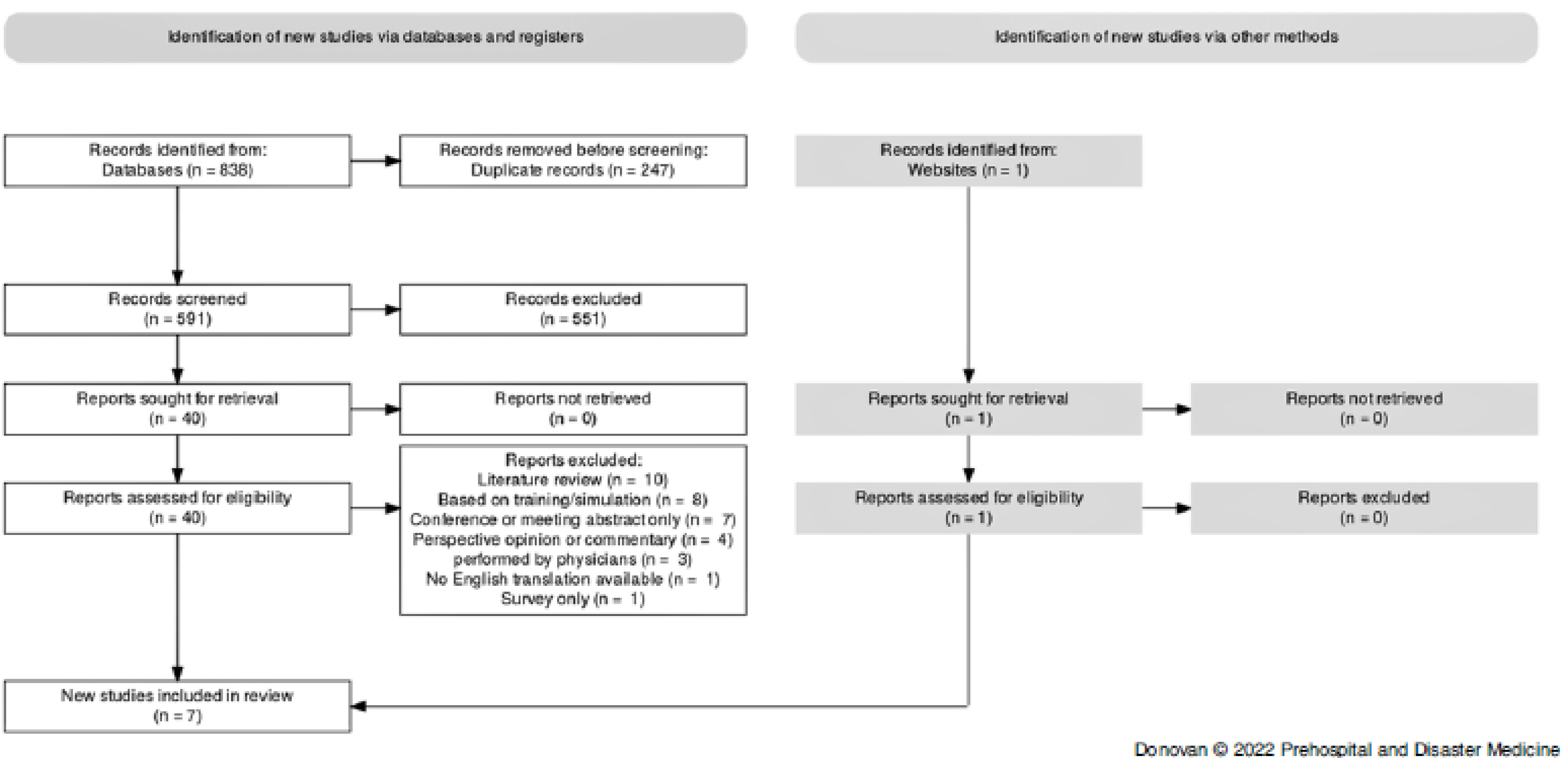
Selection Flowchart.

Studies were eligible for inclusion if they reported on lung ultrasound, the out-of-hospital setting, non-physician operators, and were published from January 1, 1990 through April 14, 2021. This period was selected because a preliminary review of the literature implied there would not be any relevant studies before 1990, as handheld ultrasound technology is relatively new. Studies were excluded if: the ultrasound was performed by physicians, the article was based on training and simulation, the article included only trauma patients, or the scan was performed in the in-hospital setting. Studies that had no English translation available were also excluded. The databases were searched by one author (JD). Following the searches, duplicates were removed and the articles were uploaded into the Covidence (Covidence systematic review software; Veritas Health Innovation; Melbourne, Australia) review application for screening. Three authors (JD, SB, and BM) screened the titles and abstracts for inclusion. Two authors (JD and SB) then sourced and reviewed the full texts of the remaining articles for relevance to the research question. Conflicts were resolved by consensus of the disagreeing parties. Following this, a search of the grey literature was conducted in Google Scholar, which identified one further study for inclusion. The selection process is shown in Figure 1.

The data were extracted from included studies per the method described by Levac, et al.^19^ The categories for extraction are author/year/country, participants/numbers, aims, methods/duration, scan protocol, and clinical indication. A total of seven articles were included, comprising three prospective observational studies, one retrospective observational trial, one prospective feasibility study, one retrospective quality control study, and one descriptive study. The results are presented below, and the summary is presented in Table 2.

**Table 2.** Study Characteristics

Author/Year/Country	Participants/Numbers	Aims	Methods/Duration	Scan Protocol	Clinical Indication
Roline^21^ 2013 USA	Unclear professional group ‘flight crew’ non-physician. Number of trained flight crew not stated. (n) = 14 patients classified as ‘medical’ rather than ‘trauma.’	To evaluate the feasibility of bedside thoracic ultrasound in the helicopter setting.	Prospective feasibility study. Duration not stated.	Two-point scan; 2^nd^ ICS anterior; Presence of sliding lung sign.	Suspected pneumothorax
Quick^22^ 2016 USA	26 flight nurses and paramedics. (n) = 13 patients classified as ‘medical’ rather than ‘trauma.’	To demonstrate the accuracy and timely detection of correctable thoracic pathology, specifically pneumothorax and improperly positioned ETTs by non-physician prehospital flight crews.	Prospective observational study. 15 months.	Continuous scan; 2^nd^ ICS - 6^th^ ICS bilaterally; Presence of sliding lung sign; ETT placement.	Suspected pneumothorax Intubated adult medical patients
Becker^25^ 2018 USA	17 paramedics. (n) = 34 patients.	To assess the feasibility of paramedic performed, remotely interpreted prehospital ultrasound in medical patients with undifferentiated respiratory distress.	Prospective observational study. 3 months.	Continuous scan; Anterior midclavicular line down the chest wall mid-to-posterior axillary line laterally/caudally; A-profile; B-profile; A/B-profile.	Objective respiratory distress /shortness of breath/SPO2 <92%
Ronaldson^23^ 2020 UK	3 advanced retrieval practitioners (nurses and paramedics). (n) = 6 patients designated as ‘medical’ rather than ‘trauma.’	To assess the feasibility of non-physicians working within a UK prehospital service to undertake prehospital ultrasound diagnosis in a live clinical environment and assess the accuracy of the pneumothorax diagnosis.	Retrospective observational study. 12 months.	Two-point scan; 2^nd^ ICS anterior; Lung slide.	Suspected pneumothorax
Schoeneck^29^ 2021 USA	22 paramedics. (n) = 65 patients.	To assess the feasibility of training paramedics in acquisition and assessment of thoracic ultrasound images in the prehospital environment.	Prospective observational study. 20 months.	Two-point scan; 2^nd^ or 3^rd^ ICS, midclavicular line; Presence or absence of B-lines.	A chief complaint of dyspnea
Pietersen^26^ 2021 Denmark	100 paramedics and EMTs. (n) = 590 scans; patient numbers not stated.	To explore the feasibility and quality of EMS personnel performed focused thoracic ultrasound.	Retrospective quality control study with a prospective gathering of data. 18 months.	Four-point scan; Anterior zone of BLUE protocol PLAPS view of BLUE protocol; B-lines; Lung sliding/lung point; Pleural effusion; Consolidation; Thickened or fragmented pleura.	Call for respiratory symptoms or symptoms suggesting lung pathology
Nadim^27^ 2021 Denmark	100 paramedics and EMTs. (n) = 771 patients.	To test the technical setup and the clinical training of the personnel, and the implementation and feasibility of the treat-and release strategy.	A descriptive study. 8 months.	Four-point scan; Anterior zone of BLUE protocol; PLAPS view of BLUE protocol; Pneumothorax; Pleural effusion; Interstitial syndrome; Lung consolidation; Other obvious abnormal finding.	Patients calling for an ambulance because of dyspnea

Abbreviations: ETT, endotracheal tube; ICS, intercostal space; EMT, emergency medical technician; EMS, Emergency Medical Services; BLUE, bedside lung ultrasound in emergencies; PLAPS, Postero-Lateral Alveolar and/or Pleural Syndrome.

## Results

The initial search found 591 articles after 247 duplicates were removed. Another 551 records were excluded as described in the study protocol. One further study was found via a search in Google Scholar. There were seven studies included in the final review, and the characteristics of these studies are presented in Table 1.

### Scan Protocol

Three out of the seven studies looked only at the identification of pneumothoraxes.^21–23^ These three studies were largely focused on trauma patients but also examined a medical cohort, thus they were included. The study by Becker, et al utilized a scan of multiple intercostal spaces (ICS) in both the anterior chest and lateral chest. This was described by the authors as “scanning down along the chest for ten seconds from the midclavicular line, followed by mid-to-posterior axillary line caudally for ten seconds.” The results of the scans were sorted into “lung profiles,” which are based on the Lichtenstein, et al bedside lung ultrasound in emergencies (BLUE) protocol.^24^ The paramedics in this study did not interpret the findings of the scans but did make a subjective assessment of “wet” versus “dry” lungs. The interpretation was made remotely by critical care trained physicians.^25^

Two studies used a similar four-point scanning protocol of anterior and lateral zones.^26,27^ Both studies looked for pneumothorax, pleural effusion, interstitial syndrome, and lung consolidation. The paramedics and emergency medical technicians (EMTs) on scene interpreted the results. One study cited the Lichtenstein, et al^24^ paper as the basis for their scan protocol. The other study cited Laursen, et al, which upon review of the article, was based on both Lichtenstein, et al and Volipicelli, et al.^24,28^ The final study by Schoeneck, et al used a two-point scan examining the second or third ICS at the midclavicular line. This article looked exclusively at B-lines and did not include an assessment for pneumothorax or pleural effusion.^29^

### Interpretation and Quality

Six out of seven studies examined paramedic/EMT direct interpretation of the scan results at the time of scanning.^21–23,26,27,29^ The study by Becker, et al had the images remotely interpreted by a physician and then retrospective assessment by an expert sonographer. Of note, 58% of scans performed by paramedics/EMTs were deemed uninterpretable by the expert sonographer.^25^ Roline, et al rated 54% of scans to rule in or rule out pneumothoraxes as “good quality” on a scale of either good or poor quality.^21^ Pietersen, et al found that the mean image quality was rated as three out of five on review by the experienced “thoracic ultrasound operator.” The agreement between the paramedics/EMTs and the expert reviewer for normal, interstitial syndrome, possible pneumothorax, and pleural effusion were presented as a Cohen’s kappa value 0.44, 0.26, 0.01, and 0.69, respectively.^26^

The study by Nadim, et al did not examine the quality of the scans in its results.

However, it is noted that “as a quality assurance measure, the stored ultrasound clips were continuously checked during the study by two of the investigators.” The authors also later characterized the scans as “adequate quality.”^27^ Schoeneck, et al found that 63% of images acquired by paramedics were adequate for interpretation. The authors compared the interpretation of paramedics with an expert sonologist (an emergency physician with ultrasound fellowship training), and the inter-rater agreement for detection of B-lines was given as a Cohen’s kappa value of 0.60. The authors compared the findings with the emergency department (ED) discharge diagnosis and so were able to provide sensitivity and specificity values for diagnosis of congestive heart failure. Bilateral B-lines had a sensitivity of 80% and a specificity of 72%, while any B-lines had a sensitivity of 93% and a specificity of 50%.^29^ Two emergency medicine consultants reviewed the scans in the Ronaldson, et al study. The reviewers rated 86.4% of the selected images to be adequate. The images were also compared with chest x-ray findings in-hospital and the expert reviewers had 100% agreement with the x-ray findings. Overall, the reported comparative sensitivity and specificity between expert reviewers and advanced retrieval practitioners was 100%. The reported sensitivity and specificity for the detection of pneumothorax was 66% and 100%, respectively.^23^ These findings are presented in Table 2.

Quick, et al compared flight nurse and flight paramedic interpretation of pneumothorax against independent “board-certified physicians” and computed tomography scan. The EMS cohort had a sensitivity of 68% and a specificity of 96% for the identification of pneumothorax, compared to an emergency physician cohort which had a sensitivity of 84% and a specificity of 98%.^22^ The Roline’s, et al study results were reviewed by an “internationally recognized expert in bedside emergency ultrasound” and compared with the interpretation of the flight crew. This study looked only at the recognition of the sliding lung sign. The expert reviewer rated 54% of images as “good quality.” The Cohen Kappa value for agreement between the expert and the EMS flight crew was 0.67, indicating substantial agreement.^21^

### Study Location

Four out of seven studies took place in the USA. Two of the studies were performed in Denmark, and one study was performed in the United Kingdom (UK).

### Indication

Three out of seven studies looked primarily at the presence or absence of pneumothorax, with one of these also listing an indication of endotracheal tube placement confirmation.^21–23^ The other four studies had variations on dyspnea or respiratory distress as their indication for scanning.^25–27,29^

### Education and Training

The education and training described in these studies ranged from 1.25 hours to six hours. The methods varied, with one study using animal models to demonstrate abnormal lung findings.^22^ Notably, one study had supervised practice in ED whilst the others used simulation or healthy volunteers.^29^ These results are presented in Table 3.

**Table 3.** Equipment, Training, and Quality

Author/Year/Country	Training/Education	Ultrasound Equipment	Quality/Accuracy of Scans
Roline^21^ 2013 USA	15-minute lecture; 60 minutes hands-on practice.	SonoSite MicroMaxx	Adequate for Interpretation = 54% of scans Sensitivity/Specificity = Not stated Cohen’s kappa = 0.67
Quick^22^ 2016 USA	Didactic series of lectures; Real-time examinations of live volunteers/live animal model to demonstrate normal and abnormal ultrasound findings. Time not specified.	SonoSite Mturbo	Adequate for Interpretation = Not stated Sensitivity/Specificity = 68/96 Cohen’s kappa = Not stated
Becker^25^ 2018 USA	30-minute lecture; 2 hours of hands-on practice. Refresher training 6 days before start of study, unspecified duration.	SonoSite iViz	Adequate for Interpretation = 41.2% of scans Sensitivity/Specificity = Not stated Cohen’s kappa = 0.385 (EMS-physician vs ED)
Ronaldson^23^ 2020 UK	No extra education, providers already performing these scans in their day-to-day practice.	GE V-Scan	Adequate for Interpretation = 86% of scans Sensitivity/Specificity = 66/100 Cohen’s kappa = Not stated
Schoeneck^29^ 2021 USA	90-minute lecture; 2-3 hours supervised practice in ED.	GE V-Scan	Adequate for Interpretation = 63% of scans Sensitivity/Specificity = 80/72 (bilateral B-lines) Sensitivity/Specificity = 93/50 (any B-lines) Cohen’s kappa = 0.60
Pietersen^26^ 2021 Denmark	4-hour lecture including hands-on training.	Phillips Lumify	Adequate for Interpretation = 74.4% of scans Sensitivity/Specificity = Not stated Cohen’s kappa: Normal – 0.44 Interstitial Syndrome – 0.26 Pleural Effusion – 0.69
Nadim^27^ 2021 Denmark	6 hours of didactic lectures and supervised hands-on training.	Phillips Lumify	Adequate for Interpretation = Not stated Sensitivity/Specificity = Not stated Cohen’s kappa = Not stated

Abbreviations: ED, emergency department; EMS, Emergency Medical Services.

## Discussion

Lung ultrasound for medical respiratory distress patients is an emerging discipline in both the in-hospital and out-of-hospital setting. Studies performed in physician-led out-of-hospital systems found that the use of lung ultrasound was feasible for various indications.^13,30^

Paramedic-performed ultrasound has previously been largely limited to traumatic indications.

This scoping review has found and examined seven studies involving lung ultrasound for “medical” patients performed by non-physicians. Notably, these studies are all less than ten years old, further reinforcing that this is an emerging area of practice for paramedics and out-of-hospital clinicians. The subgroup of studies that examined lung ultrasound by paramedics and EMTs for a broader indication of respiratory distress were all published in the past five years. The most recent studies by Pietersen, et al, Nadim, et al, and Schoeneck, et al concluded that paramedics and EMTs can be trained to use lung ultrasound and acquire images of adequate quality for interpretation when compared to experts.^26,27,29^ In contrast, an earlier study by Becker, et al found that paramedics could not acquire sufficiently useful or interpretable images and that rollout of lung ultrasound for paramedics was not feasible.^25^

A significant issue shared by most, if not all, studies identified in this review was the various barriers to enrolment or image acquisition. Many studies reported on specific reasons for the low enrolment rate. The Becker, et al study enrolled only 43.6% of eligible patients. The reasons given for not enrolling patients in this study included 25.0% equipment failure, with a further 26.5% of enrolled patients having failure of transmission.^25^ The enrolment rates published in the included studies were 43.6%-58.0% of eligible patients, with some studies not reporting on the enrolment rates at all.^25–27,29^ The reasons given included insufficient time, insufficient space, patient refusal, equipment failure, transmission failure, patient condition too critical, and no details provided. Schoeneck, et al cited low paramedic participation as a cause for low enrolments, with the alarming figure of 42% of paramedics completing only one ultrasound each.^29^ This is in keeping with the acknowledged difficulties of research in prehospital care.^31^ Conversely, the studies that included physicians did not have the same issues of low enrolments and low participation. Neesse, et al had an 86% enrolment rate and listed bright sunlight and equipment failure as reasons for lower enrolment.^13^ This highlights the need for different strategies when recruiting paramedics for participation in research.

The training and education of participating paramedics and EMTs varied considerably in the included studies. It has yet to be quantified what the ideal amount of training and education is to become competent in a particular scan for paramedic practice. A scoping review by Meadley, et al posited that “paramedics may be able to gain proficiency in POCUS reasonably promptly, regardless of base qualification, experience, duration, or perceived quality of training.”^10^ This is in contrast to physician-based EMS included in other studies where the physicians often have previous ultrasound experience in-hospital. Therefore, it may be easier for them to adopt a new scanning protocol with no need to cover the basics of ultrasound use. The difficulty in quantifying the ideal educational protocol is that the scanning protocols vary significantly from a simple bilateral check for lung slide to the more complex BLUE scan, which seeks multiple lung signs and evaluates up to eight separate zones for scanning. The Australian Society for Ultrasound in Medicine (Chatswood, Australia) requires a logbook of 25 scans and an eight-hour training course to certify competency, two hours of which must be dedicated solely to lung ultrasound.^32^ This is one available option for non-specialist physicians in Australia for credentialing in lung ultrasound. Whether this option is feasible or appropriate for paramedics is beyond the scope of this review, but it is worth considering the educational packages presented in these articles against a standard for Australian practice.

The ideal lung ultrasound protocol has not been defined for the out-of-hospital environment. A recent European Respiratory Society (Lausanne, Switzerland) publication states, “it is not possible to derive a universal and evidence-based thoracic ultrasound approach for any given clinical scenario.”^33^ This statement is reflective of the different lung protocols described by the literature. There are a variety of lung ultrasound protocols described in the literature ranging from a simple two-point anterior scan to a 28-point scan.^34^ The accuracy of these scans varies, although many have been validated in the in-hospital and out-of-hospital environment.^33^ An article by Buessler, et al compares four-, six-, eight-, and 28-point scans in the ED setting for acute heart failure and found that the six- or eight-point scan performed well against the 28-point scan. With high specificities but low sensitivities, a six-point scan is adequate for ruling out acute heart failure but less accurate at ruling in heart failure.^34^ Some of the out-of-hospital studies reference the BLUE protocol for respiratory failure. However, none of them used the full protocol as described by Lichtenstein, et al.^24^

The potential benefits of this technology are many. A significantly improved diagnostic capability in the out-of-hospital setting would be a paradigm shift in paramedic practice. There could be a significant improvement to paramedic decision making and management of this common group of patients, which in turn could improve patient outcomes. The indications for lung ultrasound in the out-of-hospital environment range from endotracheal tube confirmation to more complex lung scanning protocols assessing interstitial syndrome, pulmonary embolism, pleural effusions, and consolidation. The available literature agrees that lung ultrasound is a “simple” technique and can be easily taught.^26,30,35^ This implies that POCUS could be adapted to other settings and other professional groups.

The handheld ultrasound technology has progressed to a stage where the devices have become more affordable, increasing the rollout feasibility in the out-of-hospital setting.

## Limitations

Several limitations are shared by these studies, including low enrolments and participation as well as technical issues common in prehospital research. The seven articles that were identified were mainly of an observational study design. A lack of standardized scan protocol and training makes it challenging to evaluate the utility of this imaging modality.

## Recommendations and Conclusions

This scoping review has mapped the literature around the use of handheld ultrasound by paramedics for differentiating illnesses in respiratory distress patients. The results indicate paramedics and EMTs may be able to acquire and interpret these images compared to an expert reviewer. However, as the majority of the literature was of low quality, this study cannot draw strong conclusions about the utility of lung ultrasound for paramedics and EMTs, and more evidence is required to answer the research question. Despite the limitations, paramedics and EMTs have demonstrated the potential feasibility of lung ultrasound in out-of-hospital care. Further research should investigate the utility of standardized education and scanning protocols in paramedic-performed lung ultrasound for the differentiation of respiratory distress and the implications for patient outcomes.
